# Significance of Bishop score in single vs. double balloon catheterization for induction in labor; a meta-analysis

**DOI:** 10.3389/fsurg.2025.1584611

**Published:** 2025-12-11

**Authors:** Ping Wang, Yan Hu, Tengfei Shan, Fei Fang

**Affiliations:** Department of Obstetrics and Gynecology, Linping Campus, The Second Affiliated Hospital of Zhejiang University School of Medicine, Hangzhou, China

**Keywords:** Bishop score, single balloon catheter, double balloon catheter, labor induction, neonatal outcomes

## Abstract

**Objective:**

To conduct a meta-analysis comparing Bishop scores in newborns resulting from single balloon versus double balloon labor induction methods.

**Methods:**

We performed a meta-analysis of RCT's that compared single and double balloon catheters for inducing labor. Relevant literature studies were located by searching PubMed, MEDLINE, EMBASE, and the Cochrane Library. The primary outcome measure was the Bishop score, while secondary outcomes included the mode of delivery, complications, time interval from catheter insertion to the delivery, and the Apgar score at five minutes.

**Results:**

Our meta-analysis encompassed 6 RCT involving 996 women who underwent labor induction. We detected a substantial variation in the Bishop scores, with SBC induction yielding higher scores. Specifically, neonates delivered using SBC had higher Bishop scores than those delivered using DBC. This highlights the clinical significance of Bishop scores in identifying infants who may require immediate medical attention. However, no significant differences were found in mode of delivery, complications, or Apgar scores between the two groups (*P* > 0.05).

**Conclusion:**

Labor induction using a single balloon catheter appears to be more effective at ripening the cervix, as indicated by higher Bishop scores. This suggests that it might be a more favorable method for inducing labor.

## 1 Introduction

There is growing global concern over the rising incidence of labor inductions prior to 41 weeks, in the absence of maternal or fetal complications, due to the potential risk of iatrogenic harm to both the mother and baby (1, 2). Over the past decade, the rate of labor induction has seen a substantial rise, with elective labor inductions playing a significant role in this increasing trend (3). The CS rate continues to increase across all levels of hospitals in China (4). As the cesarean section rate rises, pregnancies involving a scarred uterus have become more frequent. A previous uterine scar heightens the risk of uterine rupture and other complications during vaginal delivery (5).

Successful labor induction is contingent upon cervical favorability. Cervical ripening modalities encompass pharmacologic agents and mechanical devices (6). The World Health Organization (WHO) has deemed mechanical ripening with balloon catheters and pharmacologic ripening with prostaglandins, including misoprostol, to be both acceptable and safe (7). Although balloon catheters and vaginal prostaglandins demonstrate similar cesarean delivery rates and maternal safety profiles in labor induction, balloon catheters are associated with a lower incidence of adverse perinatal events (8). Nonetheless, a novel meta-analysis is warranted to juxtapose the effectiveness and protection of SBC vs. DBC, given the paucity of comparative evidence currently available.

The SBC (Foley) is widely utilized mechanical technique for cervical ripening, with origins in 1960s (9). The Atad catheter, introduced in the 1990s (10), was the initial DBC variant of this device. The Cook Cervical Ripening Balloon, which received approval from the United States Food and Drug Administration in 2013, operates on the same principle as the Atad catheter. Both Foley and Atad or Cook catheters are operative mechanical means of ripening the cervix. It is claimed that, in contrast to the Foley catheter, the Atad or Cook catheter applies the dilator vector bilaterally to the cervix, thus eliminating the need for traction.

Cost-effectiveness of medical devices is a global concern, especially in settings with limited resources. In 2025, as medical technology continues to advance, there is increasing focus on the cost-effectiveness of these devices (11). We conducted a systematic review and meta-analysis to compare the efficacy and safety of two mechanical induction methods for an unfavorable cervix: SBC vs. DBC.

## 2 Materials and methods

### 2.1 Data collection

**Figure 1:**
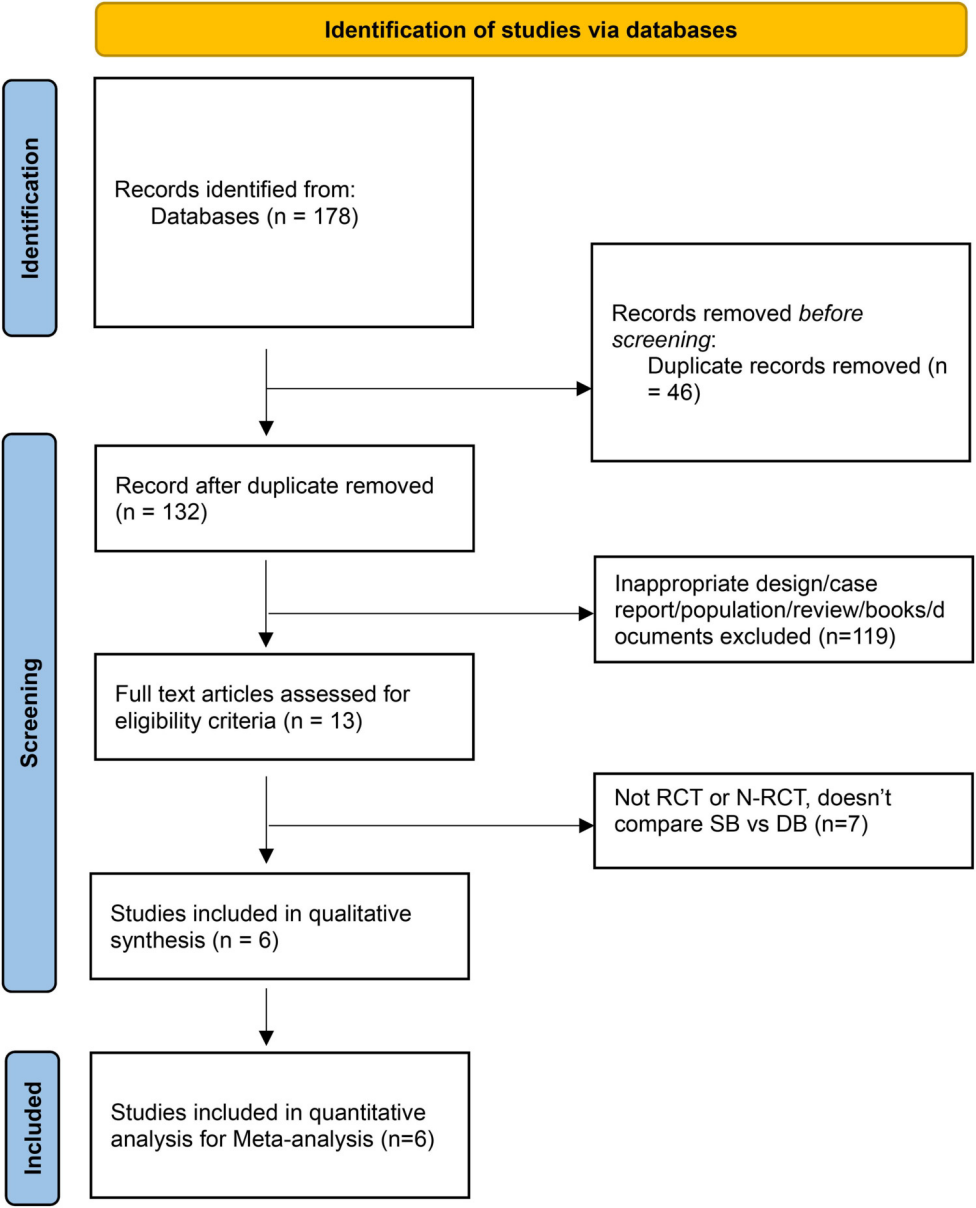
Prisma flow chart.

We performed comprehensive research of PubMed, MEDLINE, EMBASE, and the Cochrane Library, strictly following the Preferred Reporting Items for Systematic Reviews and Meta-Analyses (PRISMA) guidelines (26). Our study examines the outcomes of SBC vs. DBC labor induction, with a particular emphasis on Bishop scores, as depicted in PRISMA chart (Figure 1). This method ensures transparency and thoroughness in reporting this study.

**Figure 2:**
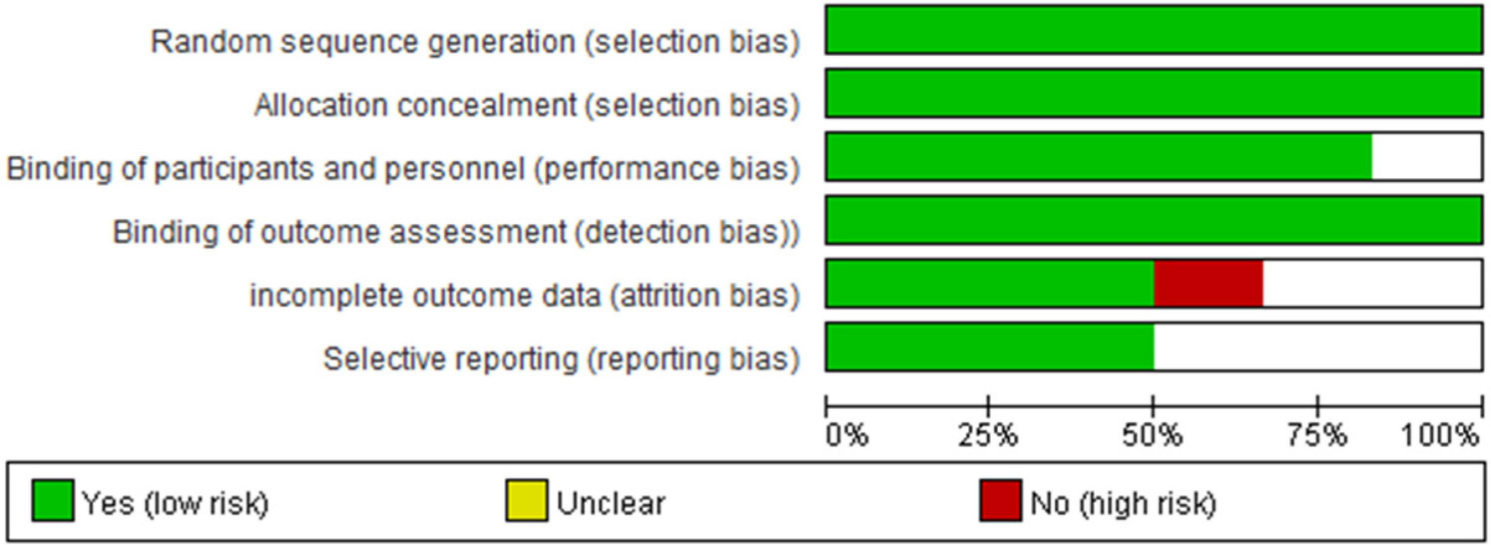
Studies bias chart.

Two reviewers independently conducted the literature search and extracted data on study design, patient recruitment, inclusion and exclusion criteria, test details, results, and quality assessment. In this analysis concentrated on outcomes associated with effectiveness and adverse events, including mode of delivery (spontaneous vaginal delivery, CS rate, and instrumental delivery), vaginal delivery in 24 h, time from catheter insertion to delivery, improvement in Bishop Score, complications during pregnancy (such as cord prolapse, placental abruption, febrile morbidity, postpartum hemorrhage, malpresentation, and maternal satisfaction), and the proportion of neonates with 5-min Apgar scores below 7. To evaluate the quality of the included RCTs, we utilized the RevMan bias chart, as illustrated in Figure 2.

### 2.2 PICO

**Population:** Articles were searched in PubMed, MEDLINE, EMBASE, and the Cochrane Library databases up to the current date (January 2025).

**Intervention:** SBC induction of labor.

**Comparison:** SBC vs. DBC induction of labor.

**Outcome:** Significant differences in Bishop score, indicating better immediate neonatal outcomes with SBC induction.

### 2.3 Inclusion criteria

Our review encompassed RCT's that compared SBC induction of labor with DBC induction in pregnant women, published up to January 2025 and accessible in full text from PubMed, MEDLINE, EMBASE, and the Cochrane Library. To be eligible, studies had to discuss the bishop score and provide data on the mode of delivery, complications, and Apgar score.

### 2.4 Exclusion criteria

To ensure the methodological soundness and relevance of the studies included in our research, we excluded non-RCT's, studies lacking applicable outcome data, those involving non-pregnant populations, studies with duplicate or overlapping data, non-comparative studies, non-human studies, and unpublished data.

### 2.5 Statistical analysis

We conducted an analysis with a 95% Confidence Interval (CI) by using RevMan 5.4 for summary statistics, which were organized based on descriptive analysis. Dichotomous data were presented as Odds Ratios (OR) with 95% CI, calculated via the Mantel-Haenszel method. Heterogeneity was evaluated using Q statistics, with total variation across studies assessed by *I*^2^, and significance set at *P* < 0.05. Heterogeneity was deemed substantial if *I*^2^ surpassed 75% and significant if *P* was less than 0.1, as determined by the *Q* test. In cases of no or minimal heterogeneity, we applied the fixed-effect model; otherwise, the random-effect model was used (12).

## 3 Results

### 3.1 Patient characteristics

Our search using the terms “single balloon,” “double balloon,” and “induction of labor” identified 178 articles from PubMed, MEDLINE, EMBASE, and the Cochrane Library. After screening the titles and abstracts, we omitted 132 articles due to duplication. We then retrieved 6 full-text articles for in-depth analysis. Of these, 119 were deemed irrelevant to our research as they did not compare single vs. double balloon induction of labor and included case reports, conference studies, literature reviews, or editorials. This process is illustrated in the PRISMA chart. The detailed characteristics of the studies are presented in Table 1.

**Table 1:** Study characteristics.

Study	Study design	Country	Patient characteristics	Age (SB/DB)	BMI (SB/DB)	Induction method	
						Single balloon (SB)	Double balloon (DB)
Pennel et al. (13)	RCT	Australia	217 nulliparous	26/27	—	Foley(16Fr)30 mL, *n* = 110	Atad device80 mL, *n* = 107
Salim et al. (14)	RCT	Israel	155 nulliparous; 138 multiparous	28.8/29.2	24.9/25	Foley(24Fr)60 mL, *n* = 145	Cook device80 mL, *n* = 148
Mei-Dan et al. (15)	RCT	Israel	92 nulliparous; 94 multiparous	29.2/29.2	29.8/27.9	Foley(16Fr)30 mL, *n* = 88	Cook device80 mL, *n* = 100
Hoppe et al. (16)	RCT	USA	50 nulliparous; 48 multiparous	29.9/30.7	—	Foley(18Fr)30 mL, *n* = 48	Cook device80 mL, *n* = 50
Pez et al. (17)	RCT	France	54 nulliparous; 23 multiparous	27.6/29.7	—	Foley(18Fr)50 mL, *n* = 45	Cook device80 mL, *n* = 32
Xing et al. (18)	RCT	China	106 nulliparous	27.4/26.7	24.2/24.9	Foley(24Fr)120 mL, *n* = 53	Cook device80 mL, *n* = 53

SB, single ballon; DB, double ballon; BMI, body mass index.

### 3.2 Mode of delivery

#### 3.2.1 Spontaneous vaginal delivery

**Figure 3:**
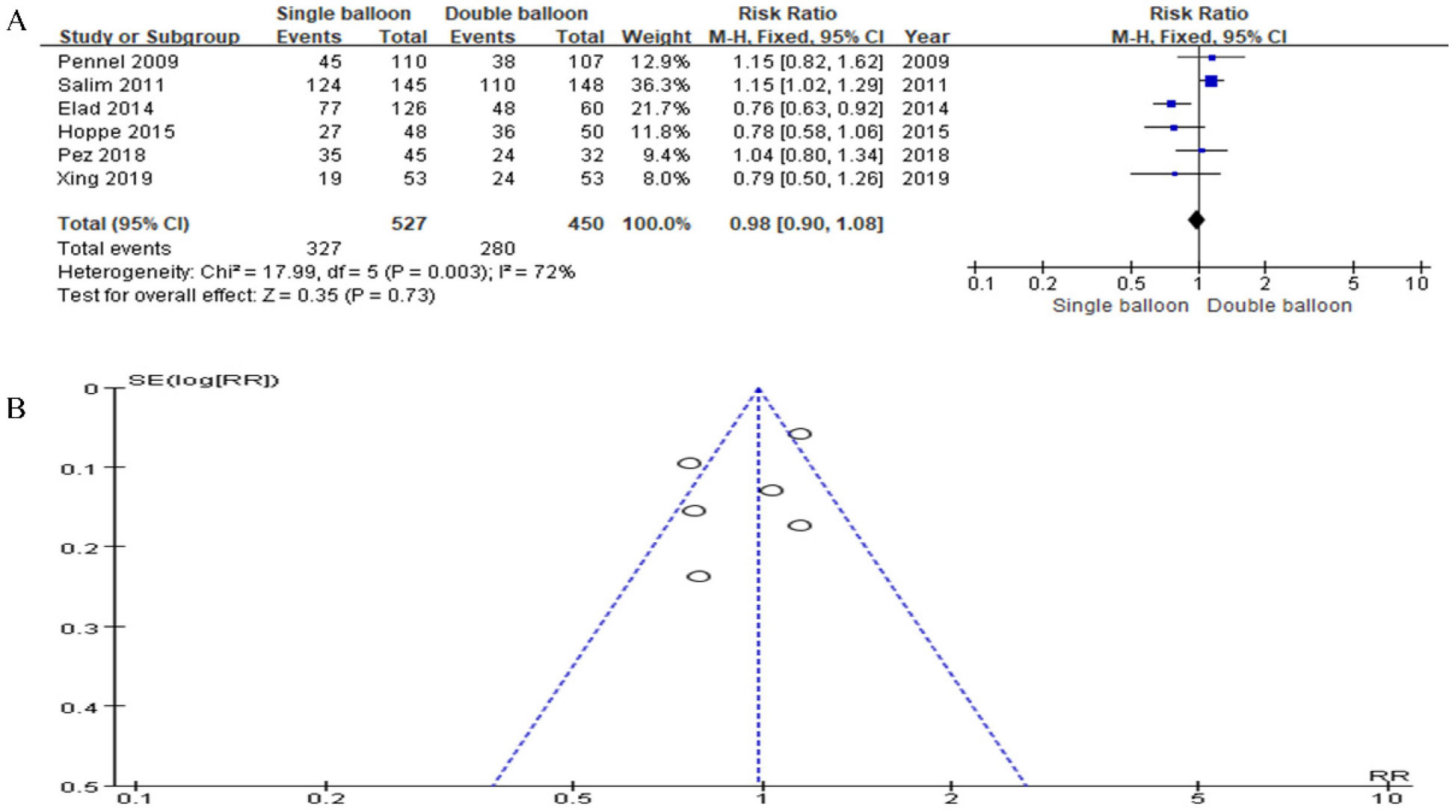
**(A)** Forest plot comparing SB and DB for spontaneous vaginal delivery (RR = 0.98, 95% CI: 0.90, 1.08, *P* = 0.73)). **(B)** A funnel plot for Spontaneous vaginal delivery.

Incorporating data from six studies into our analysis, we found that the Single Balloon (SB) group included 527 cases, while the Double Balloon (DB) group included 450 cases. The analysis revealed no significant difference in the spontaneous vaginal delivery rate between the SB and DB groups (RR = 0.98, 95% CI: 0.90, 1.08, *P* = 0.73), as depicted in Figures 3A,B.

#### 3.2.2 Instrumental vaginal delivery

**Figure 4:**
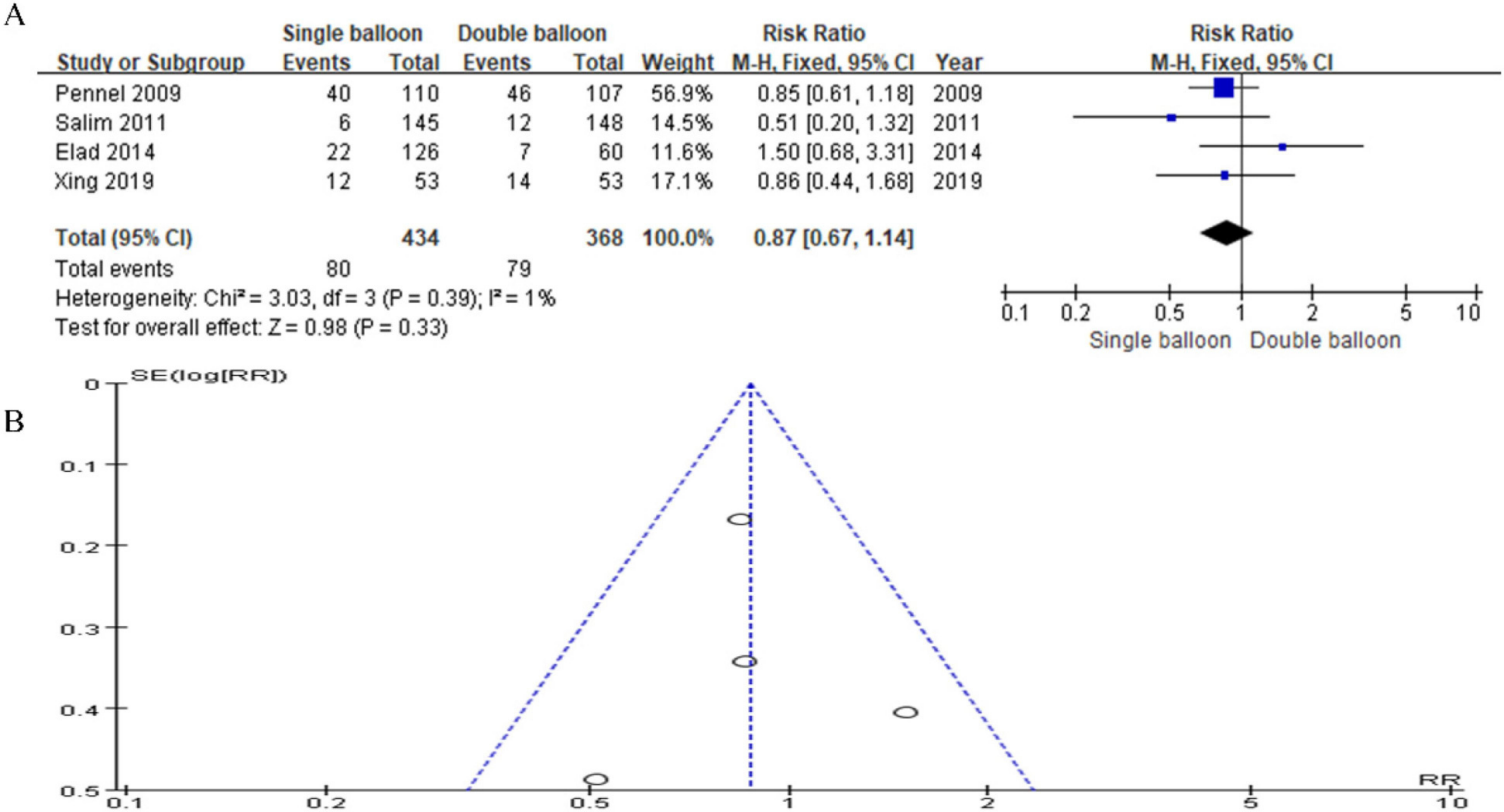
**(A)** Forest plot comparing SB and DB for instrumental vaginal delivery. (RR = 0.87, 95% CI: 0.67, 1.14, *P* = 0.33). **(B)** Funnel plot for Instrumental vaginal delivery.

In this analysis data from four articles were included. The SB group comprised 434 cases, while the DB group comprised 368 cases. The analysis did not reveal a significant difference in the instrumental vaginal delivery rate. However, the results trended slightly in favor of the SB group compared to the DB group (RR = 0.87, 95% CI: 0.67, 1.14, *P* = 0.33), as depicted in Figures 4A,B.

#### 3.2.3 Cesarean section delivery

**Figure 5:**
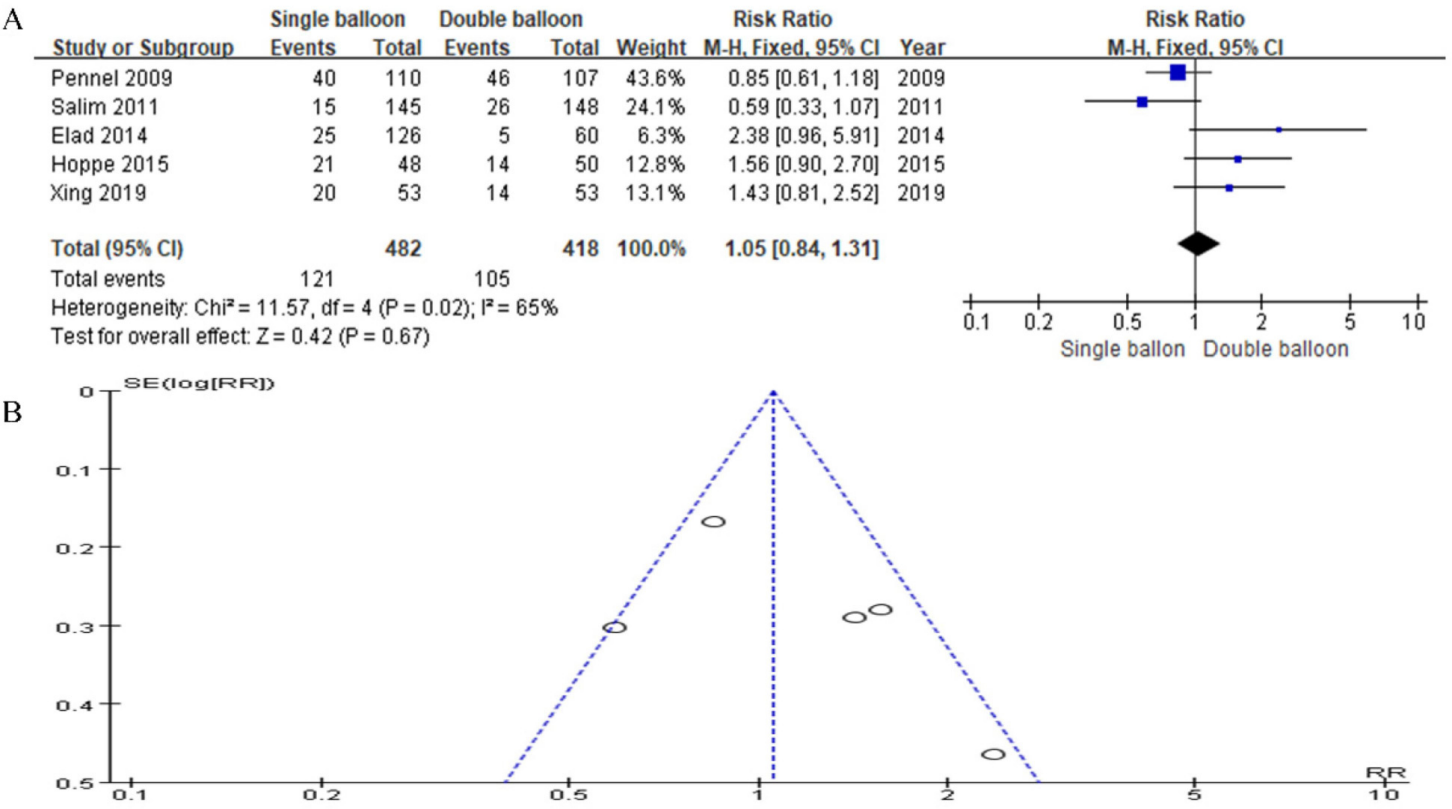
**(A)** forest plot comparing SB and DB for caesarean section delivery. (RR = 1.05, 95% CI: 0.84, 1.31, *P* = 0.67). **(B)** Funnel plot for Caesarean section delivery.

Incorporating data from five articles, our study included 482 cases in the SB group and 418 cases in the DB group. The analysis revealed no significant difference in the cesarean section delivery rate. However, the results trended slightly in favor of the DB group compared to the SB group (RR = 1.05, 95% CI: 0.84, 1.31, *P* = 0.67), as shown in Figures 5A,B.

### 3.3 Vaginal delivery <24 h

**Figure 6:**
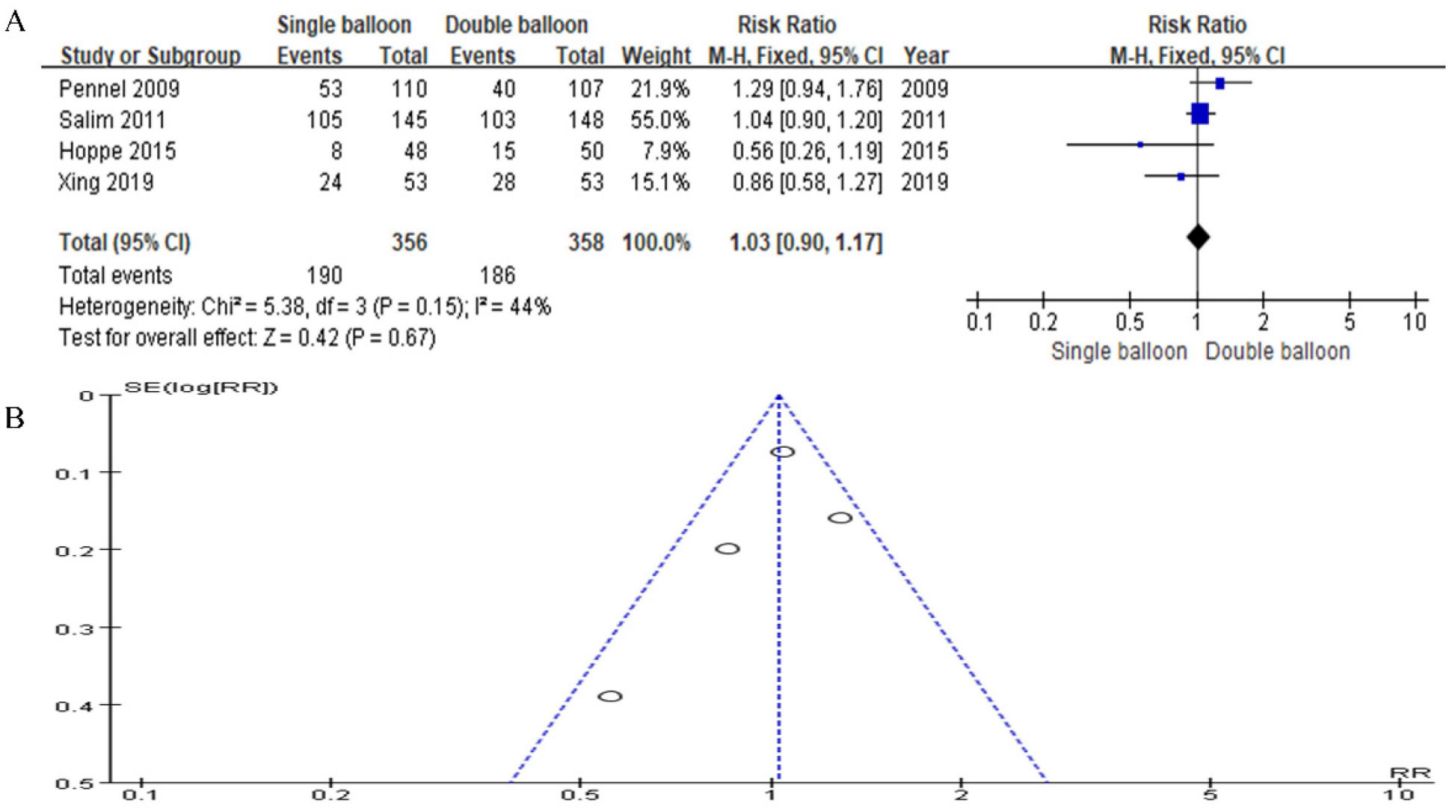
**(A)** Forest plot comparing SB and DB for vaginal delivery <24 h. (RR = 1.03, 95% CI: 0.90, 1.17, *P* = 0.67). **(B)** Funnel plot for Vaginal delivery <24 h.

In this study, data from four articles were included. The SB group comprised 356 cases, while the DB group comprised 358 cases. The analysis revealed no significant difference in the rate of vaginal delivery within 24 h (RR = 1.03, 95% CI: 0.90, 1.17, *P* = 0.67), as depicted in Figures 6A,B.

### 3.4 APGAR score at 5 min

**Figure 7:**
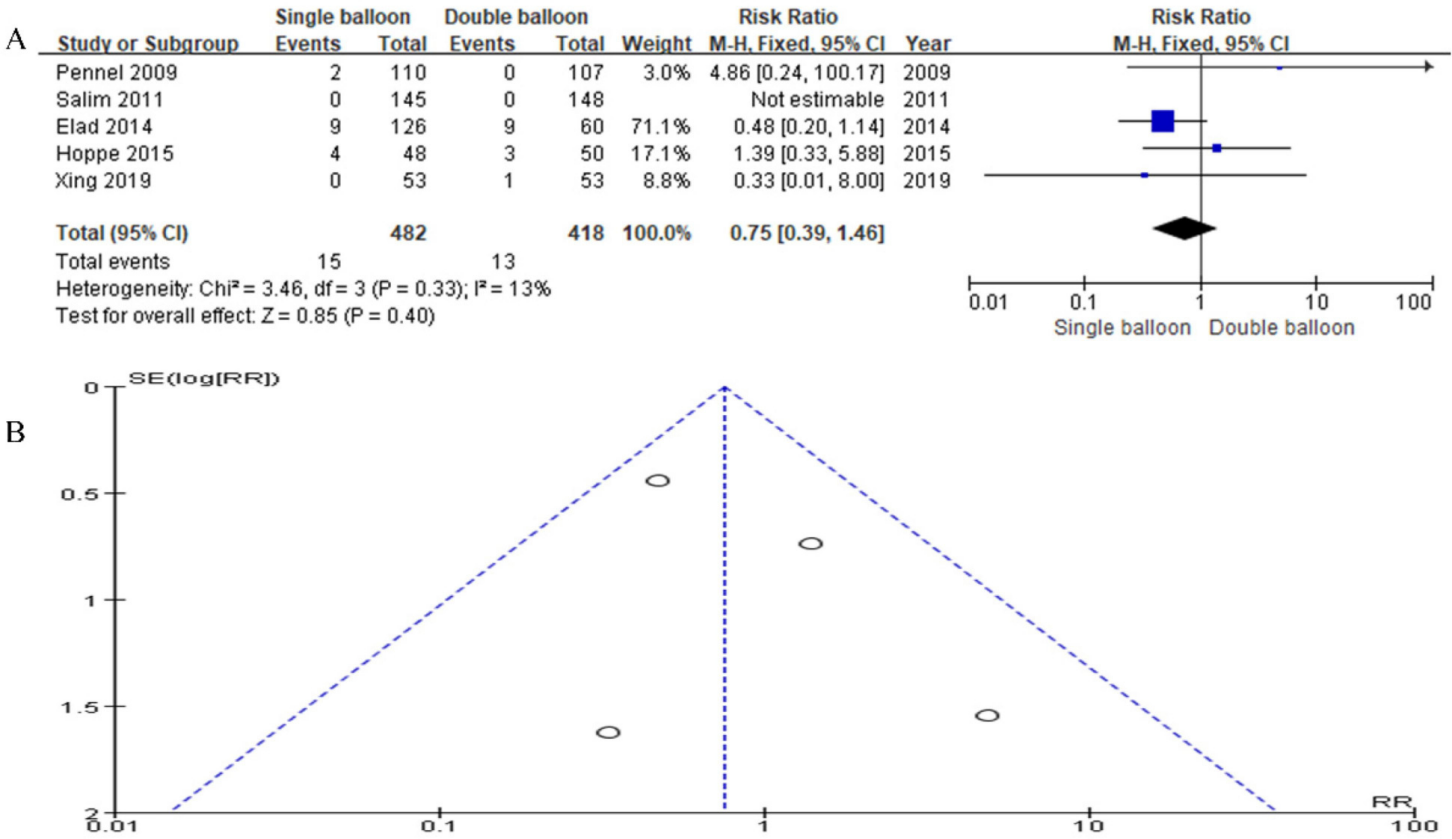
**(A)** forest plot comparing SB and DB for APGAR score at 5 min. (RR = 0.75, 95% CI: 0.39, 1.46, *P* = 0.40). **(B)** Funnel plot for APGAR score at 5 min.

Incorporating data from five articles, our study included 482 cases in the SB group and 418 cases in the DB group. The analysis revealed no significant difference in the Apgar score. However, the results trended slightly in favor of the SB group compared to the DB group (RR = 0.75, 95% CI: 0.39, 1.46, *P* = 0.40), as shown in Figures 7A,B.

### 3.5 Bishop score

**Figure 8:**
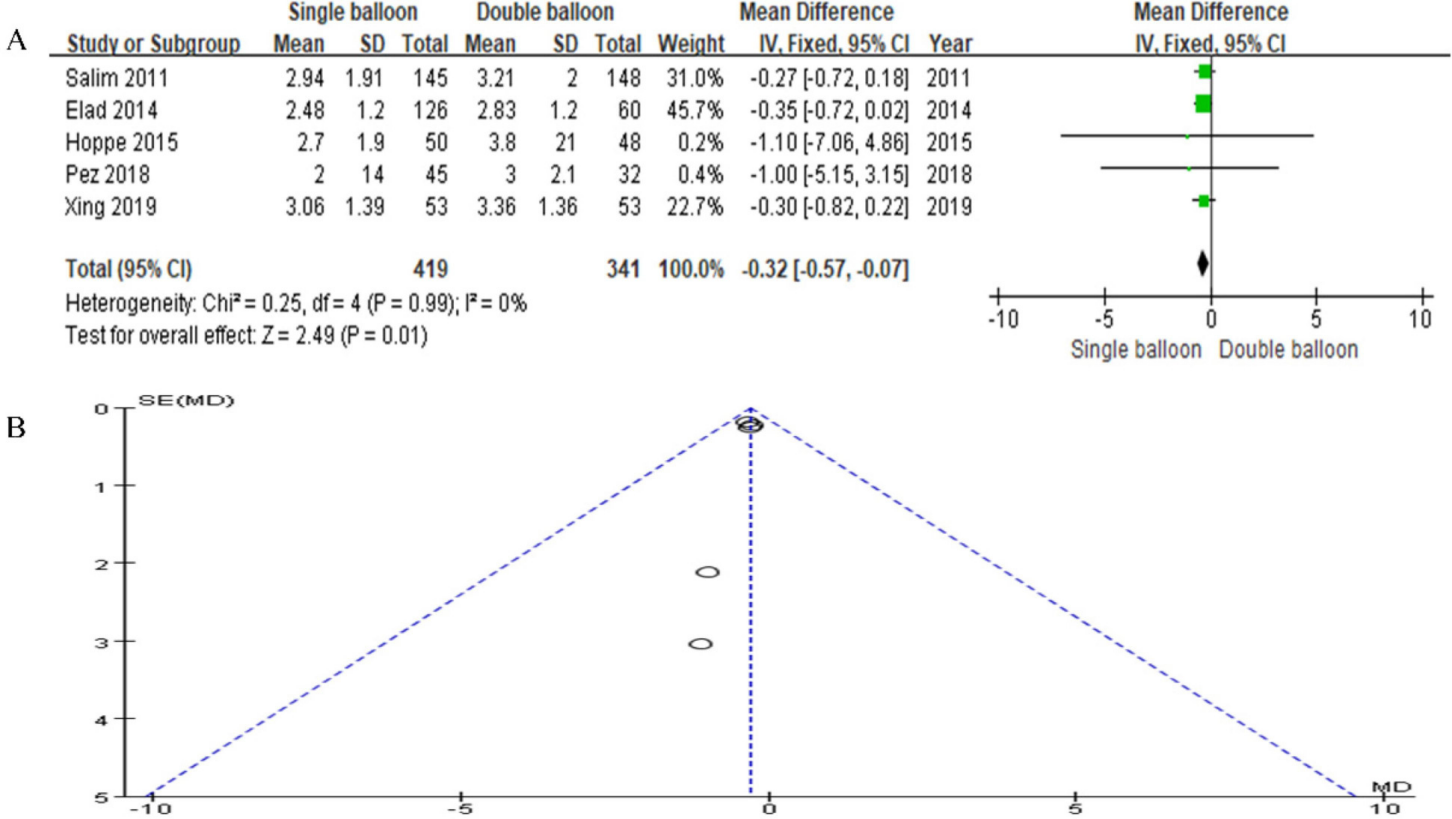
**(A)** Forest plot comparing SB and DB for bishop score. (RR = −0.32, 95% CI: −0.57, −0.07, *P* = 0.01). **(B)** Funnel plot for Bishop score.

Our study incorporated data from five articles. The SB group included 419 cases, while the DB group included 341 cases. The analysis revealed a significant difference in the Bishop score, favoring the SB group over the DB group (RR = −0.32, 95% CI: −0.57, −0.07, *P* = 0.01), as depicted in Figures 8A,B.

### 3.6 Catheter to delivery interval

**Figure 9:**
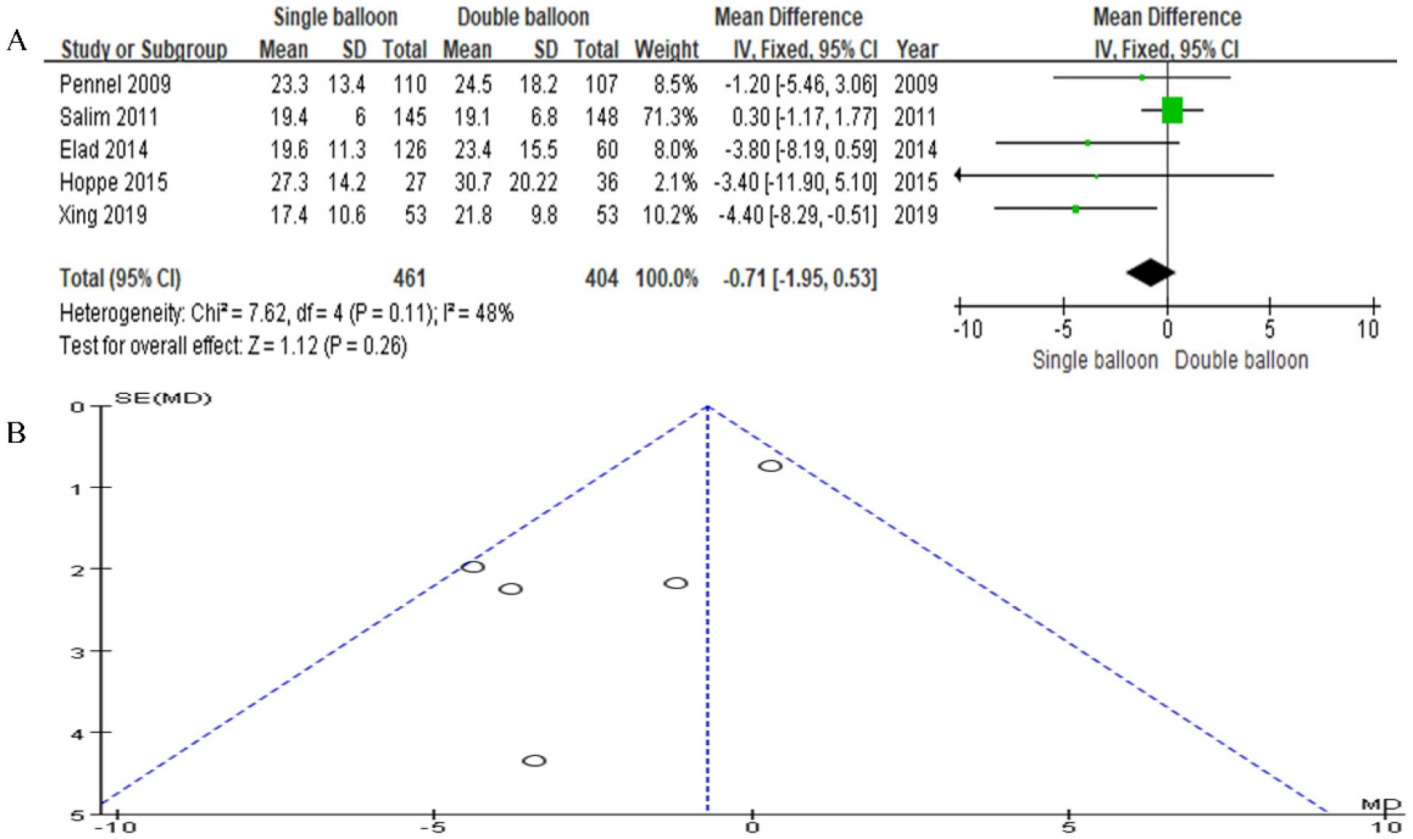
**(A)** Forest plot comparing SB and DB for catheter to delivery interval. (RR = −0.71, 95% CI: −1.95, 0.53, *P* = 0.26). **(B)** Funnel plot for Catheter to delivery interval.

Our study included data from a total of five articles. The SB group consisted of 461 cases, while the DB group consisted of 404 cases. The analysis did not show a significant difference in the catheter-to-delivery interval. However, the results trended in favor of the SB group compared to the DB group (RR = −0.71, 95% CI: −1.95, 0.53, *P* = 0.26), as illustrated in Figures 9A,B.

### 3.7 Complications

**Figure 10:**
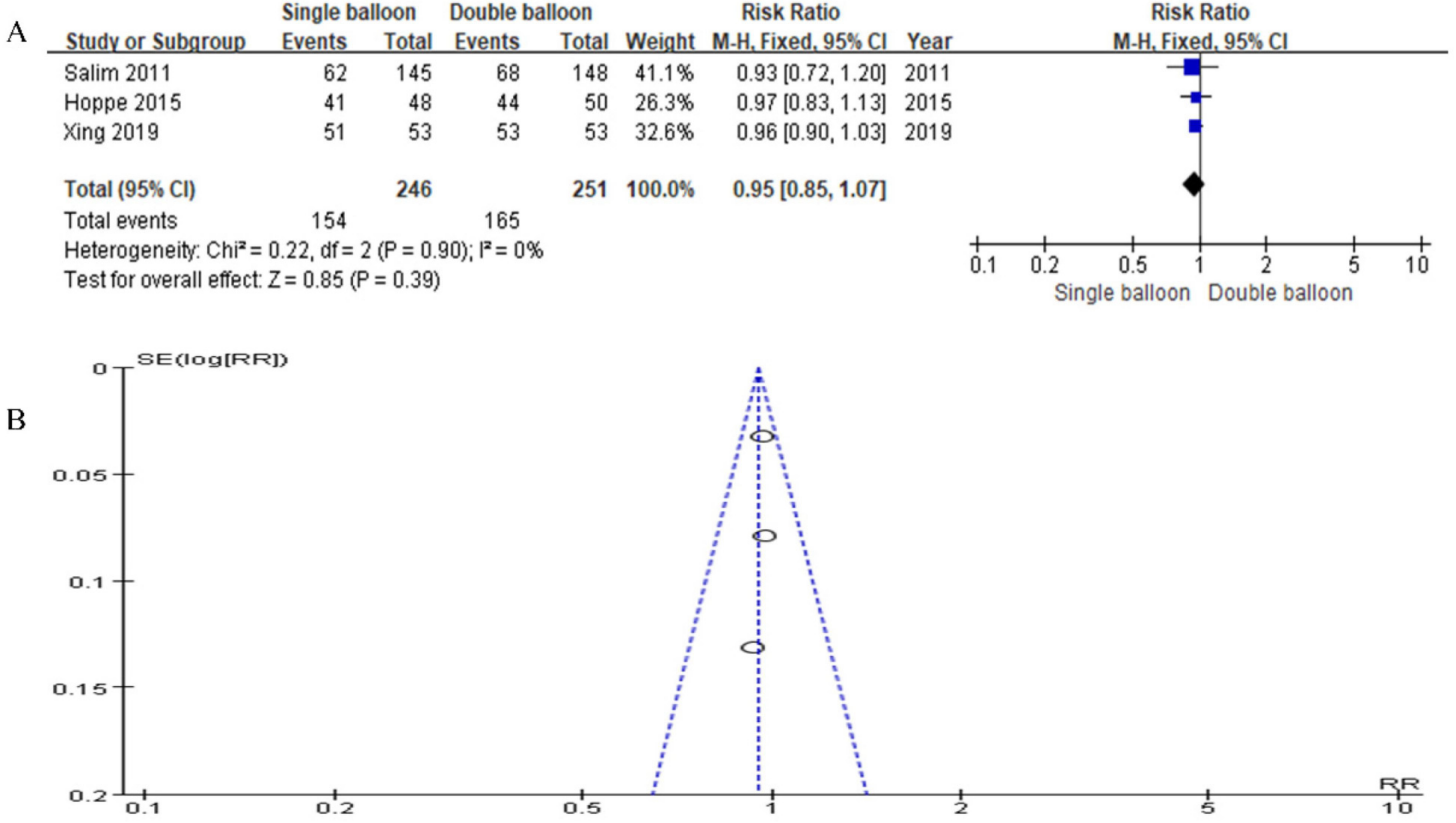
**(A)** forest plot comparing SB and DB for complications. (RR = 0.95, 95% CI: 0.85, 1.07, *P* = 0.39). **(B)** Funnel plot for complications.

Our study incorporated data from three articles in total. The SB group included 246 cases, while the DB group included 251 cases. The analysis did not show a significant difference in complications. However, the results trended slightly in favor of the SB group compared to the DB group (RR = 0.95, 95% CI: 0.85, 1.07, *P* = 0.39), as illustrated in Figures 10A,B.

## 4 Discussion

We observed in our meta-analysis a noteworthy change in the Bishop score, favoring SBC induction of labor compared to DBC induction. Specifically, the Bishop score was significantly higher in the SBC group, indicating better cervical conditions for labor induction. This finding underscores the clinical significance of the Bishop score in predicting successful labor induction and vaginal delivery. The Bishop score evaluates several clinical parameters of the cervix, including dilation, effacement, position, consistency, and the fetal head's station in the pelvis. A more favorable cervical condition for labor induction is indicated by a higher Bishop score, which can lead to a higher likelihood of successful vaginal delivery (19).

Our findings are consistent with prior studies showing that a higher Bishop score correlates with a greater likelihood of successful labor induction. For instance, a study by Erasto et al. found that oxytocin was the most effective agent for labor induction, achieving a high rate of spontaneous vaginal delivery (SVD) within a shorter timeframe (20). Similarly, a systematic review and meta-analysis by Lajusticia et al. showed that SBC induction was associated with higher Bishop scores, indicating better cervical conditions for labor induction (21). These studies emphasize the significance of the Bishop score in evaluating the effectiveness of labor induction methods (22, 23).

Our analysis did not find significant differences in Apgar scores at 1 and 5 min, mode of delivery, or complications between the two groups. This suggests that while SBC induction may lead to better cervical conditions for labor, it does not necessarily translate to improved neonatal outcomes or differences in delivery methods and complications.

In our meta-analysis, the SBC was found to be more effective in promoting cervical ripening compared to the DBC. This could be attributed to several potential mechanisms. Firstly, the SBC may exert more consistent and localized pressure on the cervix, leading to more efficient mechanical dilation. Additionally, the single-balloon design might allow for better adaptation to the individual anatomy of the cervix, thereby enhancing its effectiveness (17). Furthermore, the presence of a SB could potentially reduce the risk of infection or other complications associated with the insertion and maintenance of the device (24). Future studies should investigate these mechanisms in more detail to elucidate the specific factors contributing to the observed differences.

The meta-analysis findings suggest that SBC are more effective for cervical ripening, potentially leading to shorter labor durations and reduced need for additional interventions like oxytocin (25). These results can guide clinicians in selecting the most suitable induction method, emphasizing the importance of considering patient-specific factors such as cervical maturity and contraindications.

There are several limitations to our meta-analysis that should be taken into account when interpreting the results. For instance, the methodological quality of the included studies varied, with some studies lacking rigorous randomization or blinding procedures. Additionally, incomplete data reporting in some studies limited our ability to perform more detailed subgroup analyses.

In conclusion, our meta-analysis underscores the significance of the Bishop score in evaluating the effectiveness of labor induction methods. SBC induction appears to be more effective in ripening the cervix, as indicated by higher Bishop scores. However, future research should aim to address these limitations by conducting high-quality randomized controlled trials with standardized protocols and comprehensive data reporting. Future research should address our meta-analysis limitations through high-quality RCTs with standardized protocols, diverse patient populations, and long-term follow-up. This will enhance understanding of induction methods' mechanisms, safety, and patient satisfaction.

## 5 Conclusion

Labor induction using a SBC appears to be more effective at ripening the cervix, as indicated by higher Bishop scores. This suggests that it might be a more favorable method for inducing labor.

## Data Availability

The original contributions presented in the study are included in the article/Supplementary Material, further inquiries can be directed to the corresponding author.
